# Maprotiline Prevents Monocrotaline-Induced Pulmonary Arterial Hypertension in Rats

**DOI:** 10.3389/fphar.2018.01032

**Published:** 2018-09-21

**Authors:** Yi Tong, Qian Jiao, Yuanru Liu, Jiankun Lv, Rui Wang, Lili Zhu

**Affiliations:** Shanghai Key Laboratory of New Drug Design, School of Pharmacy, East China University of Science and Technology, Shanghai, China

**Keywords:** pulmonary arterial hypertension, soluble guanylate cyclase, maprotiline, human pulmonary artery smooth muscle cells, hypoxia

## Abstract

Pulmonary arterial hypertension (PAH) is a progressive disease caused by increased pulmonary artery pressure and pulmonary vascular resistance, eventually leading to right heart failure until death. Soluble guanylate cyclase (sGC) has been regarded as an attractive drug target in treating PAH. In this study, we discovered that maprotiline, a tetracyclic antidepressant, bound to the full-length recombinant sGC with a high affinity (*K*_D_ = 0.307 μM). Further study demonstrated that maprotiline concentration-dependently inhibited the proliferation of hypoxia-induced human pulmonary artery smooth muscle cells. Moreover, in a monocrotaline (MCT) rat model of PAH, maprotiline (ip, 10 mg/kg once daily) reduced pulmonary hypertension, inhibited the development of right ventricular hypertrophy and pathological changes of the pulmonary vascular remodeling. Taken together, our studies showed that maprotiline may contribute to attenuate disease progression of pulmonary hypertension.

## Introduction

Pulmonary arterial hypertension (PAH) is a progressive disease caused by increased pulmonary artery pressure and pulmonary vascular resistance, eventually leading to right heart failure until death (McLaughlin et al., 2018). Typical pathogenic features in PAH are endothelial cell dysfunction, abnormal vasoconstriction, remodeling of the pulmonary vessel walls accompanying proliferation of vascular smooth muscle cells, and thrombosis (Thenappan et al., 2018). The pathogenesis of PAH is complex, but multiple studies have suggested that an imbalance of vasoconstriction/vasodilation and proliferation/antiproliferation may be involved (Handler and Coghlan, 2010). Major therapeutic advances have been made in the past 20 years, with the introduction of novel compounds that target the three key pathways involved in the development and progression of PAH, including the endothelin (ET), NO, and prostacyclin (PGI_2_) pathways (Barst, 2008; Thenappan et al., 2018). Selexipag is developed by Actelion as an agonist of the prostacyclin receptor for the treatment of PAH, which leads to vasodilation in the pulmonary circulation (Sitbon and Morrell, 2012; Ghosh et al., 2016). It can be used as a vascular modulator in the development of PAH and provides symptomatic relief, but it fails to fully reverse the progression of the disease and reduce the high mortality rate (Antel et al., 2010; Galie et al., 2016). Currently, novel approaches and more effective agents are urgently needed for the treatment of PAH (Mercurio et al., 2018).

Nitric oxide, which is produced by NO synthase from L-arginine, is an important vascular modulator in the development of PAH (Ignarro et al., 1987). sGC is a key enzyme of the NO signaling cascade, which quickly attracts interests as a therapeutic target in cardiopulmonary disease (Murad, 2006; Bryan et al., 2009). NO activates sGC to catalyze the conversion of GTP to cGMP (Poulos, 2006). cGMP acts as one of the most important intracellular second messengers, which can regulate the downstream signal components of protein kinase G (PKG), cGMP-dependent of phosphodiesterase (PDE) and cGMP gating ion channels, and participates in cardiovascular system, nervous system and immune system (Rapoport and Murad, 1983; Denninger and Marletta, 1999; Munzel et al., 2003), thus evokes a series of physiological responses including smooth muscle relaxation, platelet inhibition, and vasodilation (Buechler et al., 1994; Warner et al., 1994; Murad, 2006). Ko et al. (1994) identified the indazole derivative YC-1 as the first sGC stimulator. But it has relatively weak sGC stimulating potency, moreover, it was found to inhibit phosphodiesterase and thus exerted additional cGMP-independent effects. Therefore, it was revealed that YC-1 has poor pharmacokinetic properties and lacks specificity (Mulsch et al., 1997; Hoffmann et al., 2015). In contrast to YC-1, BAY 41-2272 is a highly specific sGC stimulator and has no relevant inhibition of phosphodiesterase (Hoenicka and Schmid, 2008). Further studies led to the analog BAY 41-8543 displaying higher sGC stimulating potency, however, they both displayed low metabolic stability and low oral bioavailability in rats and showed a strong inhibition as well as induction of metabolizing cytochrome P450 (CYP) enzymes (Evgenov et al., 2006; Mittendorf et al., 2009). Afterward, riociguat was found to show no relevant CYP interaction and have a superior pharmacokinetic profile, including good oral bioavailability across different species (Mittendorf et al., 2009). Moreover, riociguat was approved for the treatment of PAH by the FDA in 2013 (Ghofrani et al., 2013). In our study, we aim to screen lead compounds targeting sGC and evaluate their effects on MCT-induced PAH model.

## Materials and Methods

### Materials

Dulbecco’s modified Eagle’s medium was purchased from Hyclone Laboratories (Logan, UT, United States). Fetal bovine serum (FBS) and dimethylsulfoxide (DMSO) were purchased from Sigma-Aldrich (St. Louis, MO, United States). 3-(4,5-Dimethylthiazol-2-yl)-2,5-diphenyl-tetrazolium bromide (MTT) and maprotiline were purchased from TCI Development Co., Ltd. (Tokyo, Japan). MCT was purchased from Biopurify Phytochemicals Ltd. (Chengdu, China) and dissolved in normal saline with 20% absolute ethanol (v/v) to a final concentration of 12 mg/mL. 50 mM stock solution of maprotiline was dissolved in DMSO, and stored at −80°C. The stock solution was diluted to the final concentrations before use, making sure that the concentration of DMSO in each well was 0.5% (v/v). The cGMP Complete ELISA Kit was obtained from Abcam (Cambridge, United Kingdom). IBMX, DEA/NO, and GTP were obtained from Sigma-Aldrich. The compound library used in this study contained 833 listed drugs. Male Sprague-Dawley rats were obtained from Shanghai Sipper-bk Laboratory Animal Co., Ltd., Shanghai, China.

### Ethics Statement

The animal experiments were conducted with the approval of the Animal Ethics Committee of East China University of Science and Technology. All the procedures related to animals’ handling and treatment were performed in compliance with the Guidelines for the Care and Use of Laboratory Animals in Shanghai, China.

### Expression and Purification of Human Recombinant sGC

The α1 and β1 subunits of human recombinant sGC were coexpressed by insect baculovirus expression system. Sf9 cells were coinfected with α1-and β1-expressing viruses, and sGC-expressing Sf9 cells were harvested 3 days after infection (Hoenicka et al., 1999; Lee et al., 2000). The cell pellets were resuspended in lysis buffer (50 mM TEA, pH 7.4, 10% glycerol, 4 mM MgCl_2_), disrupted by sonication and centrifuged at 12000 rpm for 60 min at 4°C. The supernatants were loaded to a His-Trap column, which was washed with lysis buffer containing 5 mM imidazole and eluted with a gradient from 5 to 500 mM imidazole. The fractions containing the recombinant sGC were pooled and concentrated to 200 μL in the lysis buffer containing 2 mM DTT. Then it was loaded to a DEAE column equilibrated with the buffer containing 50 mM TEA, pH 7.4, 10% glycerol, 4 mM MgCl_2_, 2 mM DTT. The bound proteins were eluted with a linear gradient of increasing ionic strength of potassium chloride in the equilibration buffer. The fractions containing the recombinant sGC were pooled, and protein concentration was determined by the method of brandford. Purified sGC was stored in aliquots at −80°C for subsequent use.

### Surface Plasmon Resonance (SPR)

Surface plasmon resonance (SPR) experiments were performed with a BIAcore T200 instrument (Thillaivinayagalingam et al., 2010). The running buffer contained 1.05 × PBS, 0.025% (v/v) surfactant P20, pH 7.4, 3 mM EDTA, 10 mM MgCl_2_, 1 mM DTT, and 1% DMSO. Purified sGC was diluted to 50 μg/mL by 10 mM sodium acetate solution (pH 4.5) containing 1 mM ATP and 1 mM DTT (Mota et al., 2014). The protein was then covalently immobilized on a CM5 sensor chip by amino coupling, and the final immobilization level was 12035 resonance units (RU). The analyte was diluted using the running buffer. All analyte measurements were performed at a flow rate of 30 μL/min and an extra wash with 10% DMSO was performed between each injection. Data processing and analysis were performed using BIA evaluation software.

### Cell Growth Inhibition Assay

Human pulmonary artery smooth muscle cells (HPASMCs) were cultured with DMEM containing 10% FBS in a humidified incubator at 37°C. The growth inhibition assay was divided into hypoxia and normoxia groups and determined by MTT method. A total of 3000 cells/well were seeded in a 96-well plate and incubated in DMEM containing 10% FBS for 24 h, then the medium was changed with serum-free medium and cells were cultured for another 24 h. Ten microliters of maprotiline or YC-1 diluted to several different concentrations were added to each well, then the cells were incubated for 48 h in a humidified hypoxic chamber set at 1% O_2_ and 5% CO_2_. Meanwhile the cells without adding compounds were incubated as control in a normoxic chamber supplied with room air and 5% CO_2._ Then 10 μL of MTT solution (5 mg/mL) were added to each well. Subsequently, the plate was incubated for 4 h at 37°C in a cell culture incubator. Afterward, the culture medium was removed and 100 μL DMSO were added to each well to dissolve the formazan crystals. Finally, the absorbance was measured at 490 nm using a Synergy^TM^ 2 multi-mode microplate readers (BioTek, United States). The final results were recorded by averaging at least three independent determinations.

### MCT-Induced PAH and Drug Treatment

Fifty six male Sprague-Dawley rats weight 180–200 g were raised at a temperature of 20–25°C with relative humidity of 50–60%, and a 12 h light/dark cycle. Food and water were available *ad libitum*. In this experiment, rats were divided into six groups: (1) the control group, (2) the model group, (3) MCT plus selexipag (po, 1 mg/kg twice daily), (4) MCT plus maprotiline (ip, 2.5 mg/kg once daily), (5) MCT plus maprotiline (ip, 5 mg/kg once daily), and (6) MCT plus maprotiline (ip, 10 mg/kg once daily). Five groups were given a single injection of MCT (60 mg/kg, sc), and one group was given saline subcutaneously and served as a control. At the day of MCT injection, selexipag, maprotiline resolved in normal saline or vehicle was administrated daily lasting for 21 days.

### Hemodynamic Measurements and Morphologic Analyses

At the end of administration, all rats were anesthetized with urethane (20%, ip). A 0.5% heparin-filled blunt-ended PE 50 catheter connected to eight-channel physiology recorder (AD Instruments, Australia) was inserted into right external jugular vein, and then positioned in the right ventricle (RV). The RVSP and mPAP were recorded. Then the lung and heart tissues were removed for the following experiments. The heart tissues were divided into right ventricle (RV) and left ventricle (LV) plus septum (S), the ratio of RV/LV + S was calculated as an index of RV hypertrophy. Right lung tissues were cut and soaked in formalin. After being fixed for 48 h, the tissues were embedded in paraffin, cut into 4-μm-sick sections and stained with haematoxylin-eosin. Structure remodeling of the pulmonary arterioles was detected using a computer-interfaced light microscope (Nikon, Japan). Pulmonary arterioles with diameter of 50–150 μm were randomly chosen for observation and analyzed by Image-Pro Plus software 6.0. For each artery, the ratio of media wall thickness (WT%) and the ratio of media wall area (WA%) were calculated as follows: (WT%) = (outside diameter–inside diameter)/(outside diameter) × 100, (WA%) = (medial wall area)/(total vessel area) × 100.

### cGMP Assay

Soluble guanylate cyclase enzymatic activity was assayed using GTP to cGMP conversion assay. Maprotiline was diluted with reaction buffer (40 mM TEA, 5 mM MgCl_2_, pH 7.4) and IBMX was dissolved in DMSO. The reaction solution contained the test compound, 2.6 mg/mL GTP, 1.1 mg/mL IBMX, and 20 μg/mL purified recombinant sGC in a volume of 10 μL (Selwood et al., 2001). The mixtures were incubated for 10 min at room temperature. The reaction was started with the addition of 90 μL of 667 nM DEA/NO. Finally, the production of cGMP was measured by the cGMP Complete ELISA Kit according to manufacturer’s instructions.

To measure the pulmonary release of cGMP, the lung tissues from rats were snap-frozen quickly after separated. A 100 mg sample of frozen tissue was homogenized in 1 mL of 0.1 M HCl. After homogenized on ice, the tissue extract was centrifuged at 12000 rpm/min for 5 min. The supernatant was collected and measured at 450 nm according to the manufacturer’s instructions of the cGMP Complete ELISA Kit.

### Statistical Analysis

Results were expressed as means ± SEM. Data were assessed with GraphPad Prism 5.0 software. Differences in measured variables between groups were determined by one-way ANOVA analysis of variance followed by the Dunnett’s Multiple Comparison Test for multiple comparisons. In all analyses, *P* < 0.05 was considered statistically significant.

## Results

### SPR-Based Compound Screening

**FIGURE 1:**
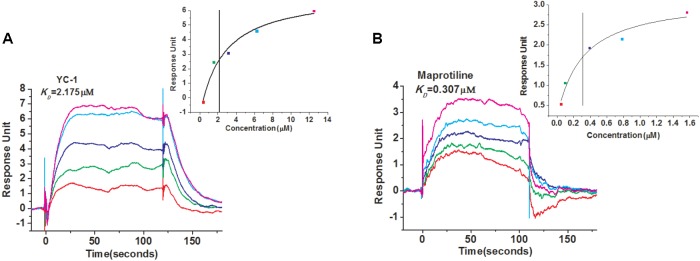
SPR evaluation of YC-1 and maprotiline binding to sGC immobilized on a sensor chip. **(A)** SPR sensorgrams of YC-1 binds to sGC with a *K*_D_ of 2.175 μM. **(B)** SPR sensorgrams of maprotiline binds to sGC with a *K*_D_ of 0.307 μM.

In order to identify new compounds binding to sGC, we performed SPR-based screening from our in-house compound library. YC-1, which is a reported compound targeting sGC, was chosen as the positive control for the binding assays. The running buffer (1.05 × PBS, 0.025% (v/v) surfactant P20, pH 7.4, 3 mM EDTA, 10 mM MgCl_2_, 1 mM DTT) was used as the negative control. Our measurement showed that the binding of YC-1 to sGC had a *K*_D_ value of 2.175 μM (**Figure 1A**), comparable to the previous reports (Purohit et al., 2014). Initially, the compounds from our library were screened at 100 μM to evaluate whether they can bind to sGC. After the initial screening, the dose-response assays were performed to calculated the *K*_D_ values. Finally, we identified that the most potent compound maprotiline bound to sGC with a *K*_D_ value of 0.307 μM (**Figure 1B**).

### Antiproliferation Activity of Maprotiline on HPASMCs

**FIGURE 2:**
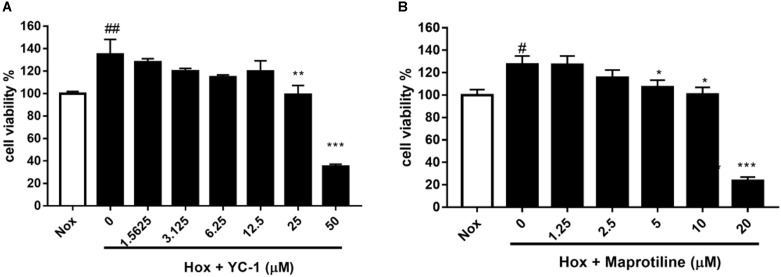
Effects of YC-1 **(A)** and maprotiline **(B)** on proliferation of HPASMCs under hypoxic conditions *in vitro*. All values represent means (± SEM) derived from duplicate samples. For statistical significance, ^#^*P* < 0.05, ^##^*P* < 0.01 vs. Nox; ^∗^*P* < 0.05, ^∗∗^*P* < 0.01, ^∗∗∗^*P* < 0.001 vs. Hox (0 μM). Each group represents three samples.

We evaluated the effects of maprotiline on the proliferation of HPASMCs under the hypoxia condition *in vitro*. MTT assays showed that the proliferation of HPASMCs was elevated under the hypoxia condition compared with the cells under the normoxic condition. However, increased cell proliferation in hypoxia group was inhibited markedly by treatment of maprotiline and YC-1. Five micromolars maprotiline significantly inhibited cell proliferation (*P* < 0.05), and 20 μM maprotiline had the greatest antiproliferative effect on HPASMCs in the hypoxic condition (*P* < 0.001). In fact, maprotiline inhibited the proliferation of hypoxia-induced HPASMCs in a concentration-dependent manner (**Figure 2**).

### *In vivo* Efficacy Studies of Maprotiline in MCT-Induced PAH Animal Model

**FIGURE 3:**
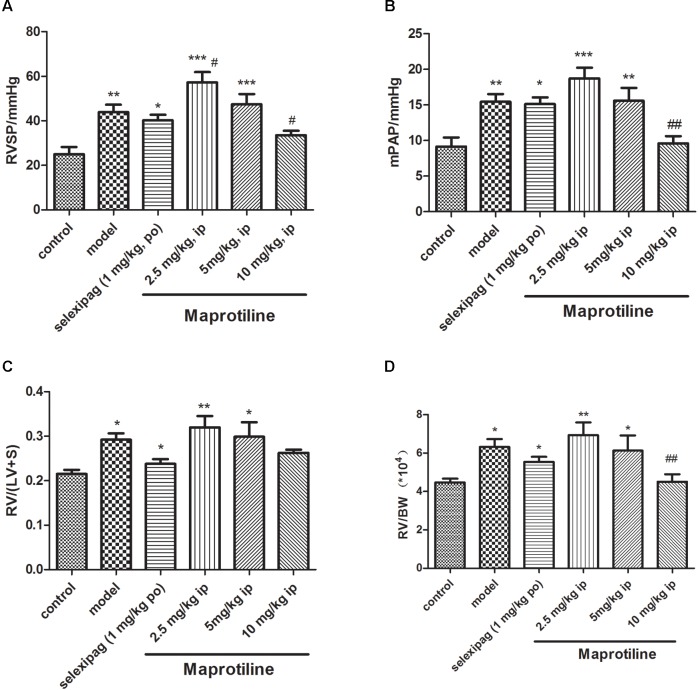
Effects of maprotiline on MCT-induced PAH animal model in SD rats. **(A)** Right ventricular systolic pressure (RVSP); **(B)** mean pulmonary arterial pressure (mPAP); **(C)** right ventricular/left ventricle + septum (RV/LV + S); **(D)** right ventricular/body weight (RV/BW) of different treatment groups. ^∗^*P* < 0.05, ^∗∗^*P* < 0.01, ^∗∗∗^*P* < 0.001 vs. control; ^#^*P* < 0.05,^##^*P* < 0.01 vs. model.

Encouraged by *in vitro* results, we next sought to evaluate the effects of maprotiline on MCT-induced PAH model. We performed different-doses treatment on MCT-induced PAH animal model. Maprotiline was administered at doses of 2.5, 5, and 10 mg/kg/day by enterocoelia. Results showed that the right ventricle systolic pressure (RVSP) of model group (43.92 ± 3.40 mmHg) increased significantly compared with control group (25.01 ± 3.21 mmHg, *P* < 0.01), which indicated the successful establishment of MCT-induced PAH model. The rats treated with low dose of maprotiline (2.5 mg/kg, 5 mg/kg) had no protective effect on MCT-induced PAH and even worsened (**Figure 3**), the reason may be due to constantly intraperitoneal injection leading to the rats in the poor state. Remarkably, intraperitoneal injection of maprotiline effectively decreased the RVSP at dosage of 10 mg/kg (33.59 ± 2.0 mmHg, *P* < 0.05 vs. model) (**Figure 3A**). Meanwhile, the mPAP of rats treated with high dosage maprotiline (9.59 ± 1.02 mmHg) was significantly reduced, compared with model group (15.45 ± 1.06 mmHg, *P* < 0.01) (**Figure 3B**). The RV/(LV + S) and RV/BW ratios were calculated to evaluate the extent of RVH. The value of RV/(LV + S) in the MCT plus maprotiline (10 mg/kg) group (0.26 ± 0.01) was relatively lower than the model group (0.29 ± 0.01) (**Figure 3C**), and this index has a downward trend with the increase of drug concentration, significant differences might be observed when the number of samples were increased. MCT injection resulted in an obvious increase of RV/BW ratio (*P* < 0.05), an index of RVH. However, the significant RVH was attenuated by administrating maprotiline at the dosage of 10 mg/kg (*P* < 0.01) (**Figure 3D** and **Supplementary Table S1**). These results indicated that administration of maprotiline (ip, 10 mg/kg once daily) could attenuate disease progression on MCT-induced rat PAH.

### Effects of Maprotiline on MCT-Induced Pulmonary Artery Morphology

**FIGURE 4:**
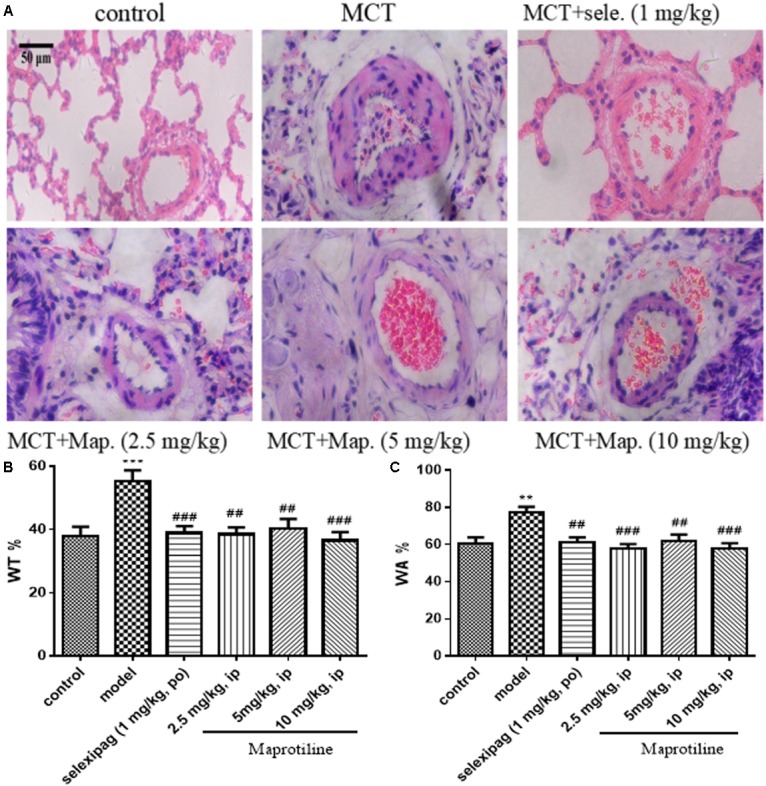
Effects of maprotiline on pulmonary vascular remodeling in MCT-induced PAH. **(A)** Representative photomicrographs of the pulmonary arterial remodeling indicated by H&E staining (Original magnification 200×, scale bars: 50 μm); **(B)** percentage of medial wall thickness (WT%); **(C)** percentage of medial wall area (WA%) of pulmonary arterioles. ^∗^*P* < 0.05, ^∗∗^*P* < 0.01, ^∗∗∗^*P* < 0.001 vs. control; ^#^*P* < 0.05,^##^*P* < 0.01,^###^*P* < 0.001 vs. model.

To further confirm the effect of maprotiline, we next performed the morphometric analysis on MCT-induced pulmonary artery. H&E staining showed that the artery lumens in rats received an injection of MCT appeared smaller compared with control group, and the pathological changes were attenuated by treatment with selexipag and all dosages of maprotiline (**Figure 4A**). WT and WA% were also evaluated to further confirm the effect of maprotiline on MCT-induced pulmonary artery remodeling. As shown in **Figure 4B**, the WT% was markedly increased in rats only received an injection of MCT (55.27 ± 3.44%) compared with control group (38.07 ± 2.81%, *P* < 0.001). By comparison, in the MCT + maprotiline (2.5, 5, and 10 mg/kg) group, WT% was significantly reduced (38.52 ± 2.19%, 40.31 ± 3.08%, 36.51 ± 2.71% and *P* < 0.01, *P* < 0.01, *P* < 0.001, respectively). Similarly, MCT significantly increased the WA% in rats (77.41 ± 2.98%), compared with the control group (60.54 ± 3.31%, *P* < 0.01). The WA% in rats treated with maprotiline was reduced (57.93 ± 2.33%, 61.83 ± 3.53%, 57.60 ± 3.23, and *P* < 0.001, *P* < 0.01, *P* < 0.001, respectively) (**Figure 4C**). The above-mentioned data indicated that injection of maprotiline (2.5, 5, and 10 mg/kg/day) effectively inhibited the MCT-induced pathological changes of the pulmonary vascular remodeling.

### Measurement of cGMP Levels

**FIGURE 5:**
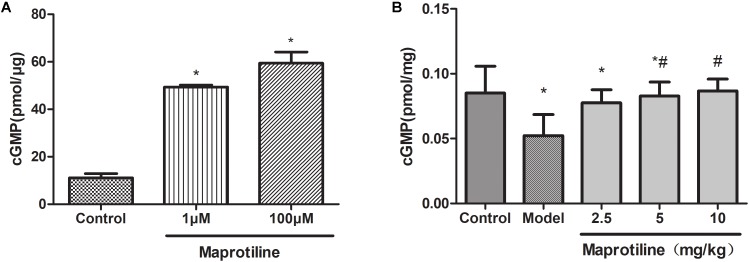
Effects of maprotiline on the enzymatic activity of sGC. **(A)** and on the production of cGMP in lung tissues. **(B)** All values represent means (± SEM) derived from duplicate samples. ^∗^*P* < 0.05 vs. control; ^#^*P* < 0.05 vs. model.

The activity of maprotiline on sGC was tested by measuring cGMP production using enzyme immunoassay system. The results indicated that maprotiline could directly stimulate sGC activity resulting in an increasing cGMP production (**Figure 5A**). In the presence of 100 μM maprotiline, the production of cGMP generated in the reaction system was 59.42 ± 8.15 pmol/μg, which was fivefold increase compared with the control (11.09 ± 3.12 pmol/μg). In addition, the effect of maprotiline on the production of cGMP in rat lung tissues was determined. Compared with the normal control group, the cGMP production in rat lung tissues was significantly reduced in the PAH model group. However, the decreased cGMP production was recovered in the treatment group with maprotiline (**Figure 5B**).

## Discussion

Pulmonary hypertension is a syndrome that encompasses several diseases, all of which have in common increased pulmonary artery pressures (Handler and Coghlan, 2010). Currently, prostacyclin, ET antagonists, and phosphodiesterase type 5 inhibitors are the primary drugs to improve the quality of life of PAH patients and alleviate symptoms (Jasinska-Stroschein and Orszulak-Michalak, 2014; Mercurio et al., 2018). However, no known agent can cure pulmonary hypertension (Antel et al., 2010). Previous studies have shown that MCT injection in rats resulted in enhanced RVSR, mPAP, RVH, increased pulmonary vessel wall thickness and PAH. These changes mainly lead to the development of PAH (Yoshiyuki et al., 2016; Karpuz et al., 2017). In this study, we used MCT-induced PAH rat model to evaluate the effect of maprotiline. Our results showed that after 21 days of single dose injection of MCT, pulmonary artery pressure was higher in the model groups compared to the control groups, and after 21 days of intraperitoneal injections of maprotiline (10 mg/kg once daily), maprotiline showed significant prevention and treatment effects on MCT-induced PAH. By analyzing the lung morphology, maprotiline was found to reduce the medial wall thickness and area of the rat pulmonary arteries induced by MCT. In conclusion, our study demonstrated that maprotiline provided protection against MCT-induced PAH in rats.

As we all know, maprotiline is a tetracyclic antidepressant with similar pharmacological properties to tricyclic antidepressants. Maprotiline inhibits neuronal norepinephrine reuptake, possesses some anticholinergic activity, and does not affect monoamine oxidase activity. Presently maprotiline is used to treat depressive affective disorders, including dysthymic disorder (depressive neurosis) and major depressive disorder. It is effective in reducing symptoms of anxiety associated with depression (Delini-Stula et al., 1995; Foye et al., 2013). Although maprotiline has multiple beneficial effects on the antidepressive system, to our knowledge, this is the first report that maprotiline can attenuate disease progression in the development of PAH. In our study, we found maprotiline could bind to sGC, a therapeutic target in cardiopulmonary disease, with a *K*_D_ value of 307 nM. Therefore, we deduced that maprotiline may directly target sGC as a regulator and thus provides protection against PAH. Previous studies revealed that the human plasma drug level of maprotiline is around 200 ng/mL (Edwards and Goldie, 1983) and binding to serum proteins is constant at 88% (Wells and Gelenberg, 1981). That is to say, a free drug concentration of maprotiline *in vivo* is about 100 nM, which is equivalent with the *K*_D_ value for maprotiline binding to sGC *in vitro* and hence gives some hope that maprotiline could be effective for human PAH. Our findings may provide important clues in identifying a new application of maprotiline in the treatment of PAH.

The most common adverse effects of maprotiline are usually drowsiness, somnolence, blurred vision, constipation, sweating, headache, arrhythmias, and memory impairment (Gareri et al., 2000). Clinical studies also reported that maprotiline might have some degree of cardiotoxicity since the significantly lengthened P-R interval and the width of the QRS complex (Hughes and Radwan, 1979). Increased white cell counts, alkaline phosphatase, decreased bilirubin, and rashes are occasionally reported in maprotiline recipients (Wells and Gelenberg, 1981). Therefore, maprotiline-based structural optimization design for the target sGC will be carried out in the future work.

## Author Contributions

LZ and RW designed the research. YT purified sGC protein and carried out the *in vitro* experiments. QJ and YL carried out the animal experiments. YT, QJ, and JL analyzed the data and draft the manuscript. LZ and RW reviewed and revised the manuscript. All authors have read and approved the final manuscript.

## Conflict of Interest Statement

The authors declare that the research was conducted in the absence of any commercial or financial relationships that could be construed as a potential conflict of interest.

## Abbreviations

HPASMCs: human pulmonary artery smooth muscle cells
MCT: monocrotaline
mPAP: mean pulmonary arterial pressure
NO: nitric oxide
PAH: pulmonary arterial hypertension
RV/BW: right ventricular/body weight
RV/LV+S: right ventricular/left ventricle + septum
RVH: right ventricular hypertrophy
RVSP: right ventricular systolic pressure
SD: Sprague-Dawley
sGC: soluble guanylate cyclase
SPR: surface plasmon resonance
WA: wall area
WT: wall thickness.
