# Supplementary material for: Maprotiline Prevents Monocrotaline-Induced Pulmonary Arterial Hypertension in Rats
Source: Front Pharmacol. 2018 Sep 21;9:1032. doi: 10.3389/fphar.2018.01032 (PMC6160570; doi:10.3389/fphar.2018.01032)
Supplement: Supplementary file 1 [file Table_1.DOCX]

Supplementary table 1. Effects of maprotiline on the RV and body weight in Sprague Dawley rats

| Group | RV (g) | Body weight (g) |
| --- | --- | --- |
| Control | 0.15 ± 0.003 | 340.80 ± 3.896 |
| Model | 0.20 ± 0.006 | 312.00 ± 3.246 |
| Selexipag (1 mg/kg) | 0.19 ± 0.003 | 304.47 ± 4.498 |
| Maprotiline (2.5 mg/kg) | 0.22 ± 0.008 | 319.30 ± 2.446 |
| Maprotiline (5 mg/kg) | 0.20 ± 0.008 | 320.18 ± 4.021 |
| Maprotiline (10 mg/kg) | 0.14 ± 0.003 | - 1. ± 3.338 |
